# Toxicological evaluation and preliminary phytochemical characterisation of a Nigerian *Cannabis sativa* chemovar

**DOI:** 10.22038/ijbms.2025.85494.18494

**Published:** 2025

**Authors:** Ebele Joan Ajagun, Babatunde Adebola Alabi, Abdul-musawwir Alli-oluwafuyi, Mary Olufunmilayo Ologe

**Affiliations:** 1 Bioresources Development Centre, Ogbomoso, Oyo State, Nigeria; 2 Department of Pharmacology and Therapeutics, College of Health Sciences, University of Ilorin, Ilorin, Nigeria; 3 Department of Pharmacology and Therapeutics, Faculty of Basic Clinical Sciences, Bowen University, Ogbomoso, Oyo State, Nigeria; 4 Pan African Cancer Research Institute (PACRI), Faculty of Health Sciences, University of Pretoria, Pretoria, South Africa

**Keywords:** Acute toxicity, Cannabinoids, Cannabis sativa, Profile, Sub-acute toxicity

## Abstract

**Objective(s):**

Different *Cannabis sativa* chemovars produce diverse pharmacological and behavioral effects. With the widespread use of cannabis in Nigeria, detailed toxicological effects of Nigerian chemovars are lacking. This study aimed to identify phytocannabinoids and investigate the toxic effects of an indigenous *C. sativa*.

**Materials and Methods:**

The plant samples were air-dried, powdered, extracted with ethanol, and characterized (phytochemical screening, Fourier Transformed Infrared Spectroscopy (FTIR), and Gas Chromatography-Mass Spectrometry (GC-MS)). Acute and subacute toxicity tests were done following Organisation for Economic Co-operation and Development (OECD) protocols.

**Results:**

Screening showed appreciable levels of alkaloids, tannins, saponins, cardiac glycosides, and phenol. FTIR analysis indicated functional groups and chemical linkages like alcohols, fatty acids, alkynes, ketones, and esters, and 11 phytocannabinoids with delta-9-tetrahydrocannabinol in abundance (35.78%) reported by GC-MS. Acute toxicity test indicated an oral lethal dose (LD_50_) value of ˃5000 mg/kg, a no-observed-adverse-effect-level (NOAEL) dose of ≤300 mg/kg, and a significant (*P<*0.05) decrease in the weight of animals in the 2000 mg/kg treatment group. The sub-acute toxicity test showed significantly (*P<*0.05) decreased ALP and ALT levels at 25 mg/kg body weight, and significantly lower triglyceride (*P<*0.01) and LDL (*P<*0.05) levels. Urea and some haematological parameters were significantly (*P<*0.05) higher in the 250 mg/kg group. Also, we observed mild to moderate necrosis in the excised pancreas and liver, and mild tubular changes in the kidney.

**Conclusion:**

This suggests that our indigenous variety of *C. sativa* may be considered safe following oral consumption.

## Introduction

*Cannabis sativa* L., an annual dioecious herb of the family Cannabaceae, is known globally as marijuana or Indian hemp and locally in Nigeria as “Igbo,” “wiwi,” and “ebo”. Native to Central Asia, it is now cultivated worldwide (1, 2) and used recreationally and medicinally for its phytoconstituents and cannabinoids, with applications ranging from analgesic to anti-inflammatory effects (2-4). Commonly smoked, it is also brewed as tea, infused in alcohol, added to food, or eaten fresh (5, 6). Nigeria ranks among the largest producers, suppliers, and consumers in West Africa (7-9). Despite its illegal status, documented use persists (10, 11). Globally, 147 million people (2.5% of the population) use cannabis, with an estimated 50,000-100,000 diabetic patients consuming it, rising from 1.7% in 2005 to 5.8% in 2018, with an unknown number self-medicating (12, 13). While bioactive plants are well represented in orthodox medicine, data on the safety and phytochemical profile of locally cultivated cannabis remain scarce. Most toxicity studies have focused on isolated cannabinoids, synthetic derivatives, or certified extracts (14), leaving a gap in knowledge regarding repeated oral administration of the crude extract. This study fills that gap by identifying the phytoconstituents and cannabinoids of an indigenous Nigerian variety and assessing its toxic effects in Wistar rats to guide dosing for future efficacy studies and evaluate potential histopathological changes in vital organs.

## Methods

### Collection, identification, and extraction of plant materials

Permission to handle *C. sativa* was granted by the National Drug Law Enforcement Agency (NDLEA), Abuja, Nigeria. Seized samples were collected from the Oyo State Command Office in Ibadan in April 2022. Authenticity was confirmed at the Department of Plant Biology, University of Ilorin, and a voucher specimen was deposited (UILH/001/1467/2023). Hand-picked leaves were air-dried, ground with a mortar and pestle, and 1000 g was extracted in 70% ethanol by cold maceration for 72 hr (15). The extract was filtered, concentrated, and the percentage yield was calculated.

% yield=weight of plant extract/weight of powdered plant material)×100

### Preliminary phytochemical tests

Fresh 70% ethanol crude extract was qualitatively analysed for secondary metabolites using standard procedures (16, 17).

### Fourier transform infrared spectroscopy (FTIR)

FTIR analysis was performed on the ethanol extract using a SHIMADZU FTIR8400S with ATR sampling (18). One mg extract was mixed with 50 mg FTIR grade KBr, compressed into a pellet, and scanned at 400-4000 cm⁻¹, resolution 4 cm⁻¹.

### Gas chromatography-mass spectrometry (GC-MS)

GC-MS analysis (SHIMADZU GC-MSTQ8050NX) on the ethanolic crude extract of *C. sativa* used an Elite5MS capillary column (30 m×250 µm×0.25 µm)(19). Helium carrier gas flowed at 1.61 ml/min. Oven temperature: 50 ^°^C (3 min), raised 10 ^°^C/min to 280 ^°^C, final 300 ^°^C. Electron ionisation: 70 eV, scan 0.3 sec, m/z 40-600. Injection volume: 0.5 µl, split ratio 20:1, injector at 280 ^°^C. Compounds were identified by retention time, peak area, and spectra compared with the NIST library (NIST11.1L).

### Experimental animals

Adult female Wistar rats (180-250 g) were used, cared for per international guidelines. Ethical approval: University of Ilorin (UERC/ASN/2023/2573).

### Acute toxicity test

Following OECD guideline 420 (17), 18 overnight fasted rats were assigned to six groups (n=3). Controls received virgin coconut oil (5 ml/kg); treatment groups received 50, 300, 2000, 3000, or 5000 mg/kg extract. Animals were monitored continuously for four hours, then twice daily for 14 days. Body weights were recorded weekly.

### Sub-acute toxicity test

Following OECD 407 (17), 24 female rats (200-250 g) were allocated to four groups (n=6): 

Virgin coconut oil (5 ml/kg), *C. sativa* (25 mg/kg), *C. sativa* (125 mg/kg), and *C. sativa* (250 mg/kg) groups. Doses were given daily by oral gavage for 28 days. Animals were monitored twice daily, and body weights were taken on day 0 and weekly thereafter. After an overnight fast, rats were weighed and euthanised on day 29. Blood was collected via cardiac puncture for biochemical and haematological analysis. Heart, brain, liver, kidneys, and pancreas were excised, rinsed, weighed, trimmed, and fixed in formalin for histology.

### Statistical analysis

Data were analyzed by one-way ANOVA (GraphPad Prism 9.02) with Dunnett’s *post hoc* test; significance set at *P*<0.05 and *P*<0.01. Results are expressed as mean±SEM.

## Results

### Extraction yield

Ethanolic extraction of *C. sativa* leaves produced a 6.3% yield (63 g of dried extract).

### Preliminary phytochemical screening

The extract contained high levels (+++) of alkaloids, tannins, saponins, phenol, and cardiac glycosides; moderate amounts (++) of phlobatannin, flavonoids, anthraquinones, and steroids; minute amounts (+) of terpenoids, while cardenolides and chalcones were absent (Table 1).

### Fourier transform infrared (FTIR) analysis

**Figure 1:**
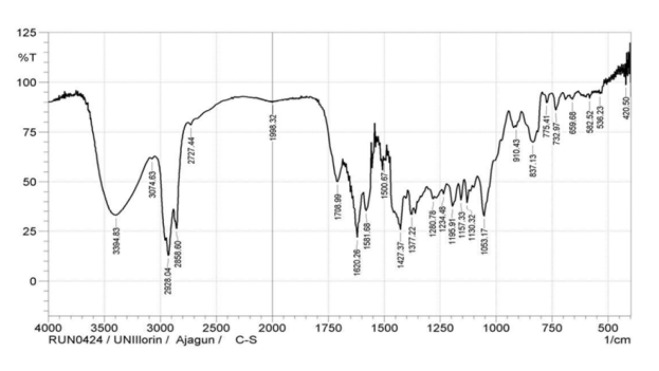
Fourier transformed infrared spectroscopy (FTIR) spectra of ethanol extract of *Cannabis sativa* leaves showing major functional group assignments

The FTIR spectrum (Figure 1) revealed prominent peaks for O-H (3394.83 cm⁻¹), aliphatic C-H stretching (2928.04 cm⁻¹, 2858.60 cm⁻¹), alkene C=C-C (1708.99 cm⁻¹), ester C=O (1620.26 cm⁻¹), and CH₃ of methylene (1053.17 cm⁻¹), reflecting a diversity of functional groups. 

### Gas chromatography–mass spectrometry (GC-MS) profiling

Thirty-nine compounds were identified, including 15 phytocannabinoids (74.64 % total peak area). Major cannabinoids were Δ⁹tetrahydrocannabinol (THC, 35.78 %), cannabigerol (9.70 %), cannabinol (5.71 %), cannabichromene (4.77 %), and cannabidiol (3.92 %). Trace components included methoxyTHC (0.20 %) and cannabicyclol (0.95 %)(Table 2). The complete list of all compounds is contained in the supplementary Table (T1). 

### Acute toxicity

**Figure 2:**
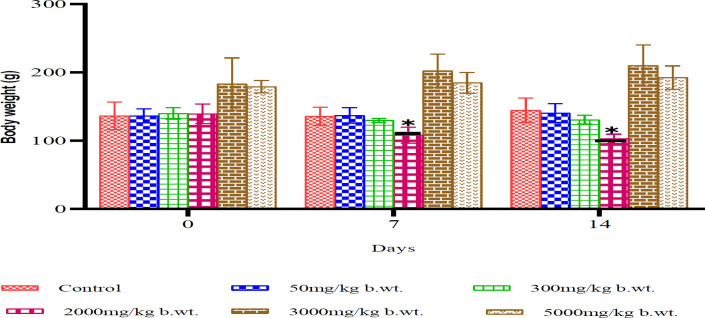
Body weight changes in rats over 14 days following acute oral administration of *Cannabis sativa* extract (mean±SD, n=3; *P<*0.05 vs day 0)

Acute toxicity testing revealed no mortality in any treatment groups, even at the highest dose of 5000 mg/kg, indicating an LD₅₀ greater than 5000 mg/kg. However, behavioral changes were observed at doses as low as 300 mg/kg, including decreased motor activity and weight loss. The 2000 mg/kg group showed a statistically significant reduction in body weight on days 7 and 14 (*P*<0.05)(Figure 2).

### Sub-acute toxicity

**Figure 3:**
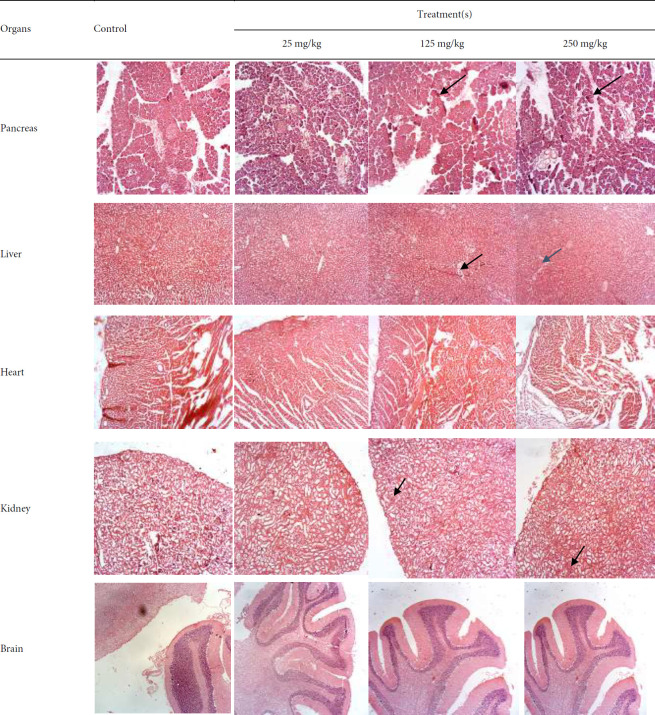
Representative histological sections of pancreas, liver, and kidney from rats treated with *Cannabis sativa* extract for 28 days, showing dose-dependent necrosis and tubular alterations (H&E stain)

Sub-acute toxicity screening over 28 days revealed dose-dependent effects. Rats in the 250 mg/kg group showed significant weight loss (*P*<0.05), as relative and absolute pancreas weights were significantly reduced in the 125 mg/kg group (*P*<0.01) relative to control. Biochemical analysis showed reduced ALP and ALT levels at 25 and 250 mg/kg, while AST levels increased at 25 and 125 mg/kg. Urea and glucose levels were elevated at higher doses, and LDH increased across all treatment groups. CK levels decreased significantly at 125 and 250 mg/kg. Lipid profile analysis showed reduced triglycerides and LDL at 250 mg/kg. Haematological analysis showed increased packed cell volume (PCV) at 250 mg/kg and elevated hemoglobin and platelet counts at 125 mg/kg. Mean corpuscular hemoglobin concentration (MCHC) decreased across all doses. White blood cell (WBC) and lymphocyte counts were significantly reduced at 125 and 250 mg/kg, while neutrophil and monocyte counts increased in all treated groups (Table 3). Histopathological examination revealed dose-dependent necrosis in the pancreas, ranging from mild (25 mg/kg) to moderate-severe (250 mg/kg). Mild necrosis was also observed in liver tissues, and mild tubular changes were noted in the kidneys across all treatment groups. No histological changes were observed in the brain or heart (Figure 3).

## Discussion

The global rise in cannabis use is especially notable in Nigeria, where our unique local variety is gaining popularity (9, 20). This rise in traditional oral consumption–whether as tea, infusions in alcoholic drinks, or as condiments and vegetables in soups– along with the use of crude plant extracts instead of pharmaceutical-grade products, influenced the decision to use crude ethanol extract and the oral route for this study (6, 9). However, there is limited data on its phytocannabinoids and the toxic effects of repeated oral intake.

The preliminary and FTIR analysis of the ethanol extract from *C. sativa* leaves confirmed the presence of several secondary metabolites, consistent with similar studies highlighting the complex nature and therapeutic effects (1, 6). The high THC levels detected by GC-MS analysis, with a (THC+CBN/CBD) ratio of >1 and a CBD/THC ratio of <0.5 (0.11), classify this local cannabis as chemotype 1 and drug-type (narcotic) according to chemotaxonomic classification (6, 21-23), suggesting a potential for toxicity. Therefore, classifying cannabis chemotypes can help predict both therapeutic and adverse effects. The observed THC levels are consistent with other studies that reported elevated levels in cannabis products, which might be due to the degradation of tetrahydrocannabinolic acid (THCA) and influenced by regional climate.

The acute toxicity profile showed effects varied with dose, with high doses causing excitatory effects, while inhibitory effects occurred at lower doses. These findings align with the literature, which emphasizes the plant’s complexity, making classification as a stimulant or depressant difficult (24, 25), with effects varying by dose, strain, and genetic factors. The lethal dose 50 (LD_50_) of *C. sativa*, which exceeds 5000 mg/kg after oral consumption, is regarded as safe according to established standards for chemical substances (17).

Subacute toxicity studies are crucial for predicting the safety of agents administered repeatedly, as they evaluate the structural integrity and function of organs through biochemical analysis (17). The observed dose-dependent weight loss agrees with other studies that report reduced body mass index (BMI) and lower rates of obesity in cannabis users (26, 27), thereby increasing interest in its potential for weight management. However, further research is necessary to explore the long-term effects on appetite-regulating hormones.

Repeated administration of *C. sativa *extract did not significantly alter liver function markers, as the observed reductions in ALP and ALT levels and increase in AST values remained within accepted ranges (28, 29). However, the mild necrosis observed in liver tissue samples across treatment groups may suggest limited safety after repeated exposure. 

The elevated plasma urea levels in animals receiving 250 mg/kg could result from dehydration, as histological examination showed mild tubular changes. The significant increase in glucose levels in the 125 and 250 mg/kg groups remained within normal ranges (30, 31). 

The toxic effects of xenobiotics and chemicals on cardiac and skeletal muscle membranes can be assessed by plasma levels of creatine kinase (CK) and lactate dehydrogenase (LDH), which act as biomarkers of muscle damage because they cannot cross the sarcoplasmic membrane (32). The observed increase in LDH levels and decreased CK levels suggests toxic effects on muscles and tissues, indicating potential damage to the organs.  

The impact of crude plant extracts on the lipid profile after oral administration is essential for predicting cardiovascular risk, with the subacute use of *C. sativa* crude extract resulting in decreased LDL and triglyceride levels in the 250 mg/kg treatment group, with LDL levels within the optimised range for female Wistar rats (31, 33). 

Blood parameters, including hemoglobin, haematocrit, red blood cells (RBC), and white blood cells (WBC), reflect health status with deviations indicating toxicity or disease conditions (28, 34). Repeated administration of *C. sativa* crude extract led to increases in PCV, hemoglobin, mean corpuscular hemoglobin concentration, and neutrophil levels, suggesting erythrocytosis (30, 31, 34). 

Changes in organ weight indicate toxicity, which may result from damage, enzyme disruption, or physiological disturbances, and are confirmed through gross examinations, clinical evaluations, and histopathological analyses (17). Mild to moderate changes in the structure of the pancreas, kidney, and liver confirm the non-lethal toxicity profile of the *C. sativa* crude extract. However, the elevated levels of some observed parameters highlight the importance of regular monitoring of individuals using *C. sativa* over an extended period.

**Table 1:** Preliminary phytochemical profile of ethanol crude leaf extract of *Cannabis sativa*

Constituent	Relative abundance
Alkaloids	+++
Tannins	+++
Saponins	+++
Phenol	+++
Cardiac glycosides	+++
Phlobatannins	++
Flavonoids	++
Anthraquinones	++
Steroids	++
Terpenoids	+
Cardenolides	−
Chalcones	−
	

**Table 2:** Major phytocannabinoids of *Cannabis sativa* identified by gas chromatography-mass spectrometry (GC-MS)

Compound	Retention time (min)	Peak area (%)
Δ⁹Tetrahydrocannabinol (THC)	23.063	35.78
Cannabigerol (CBG)	23.450	9.70
Cannabinol (CBN)	23.572	5.71
Cannabichromene (CBC)	22.169	4.77
Cannabidiol (CBD)	22.125	3.92
Others (trace cannabinoids)	20.166-25.240	≤ 2.0 each

**Table 3:** Summary of significant sub-acute effects of *Cannabis sativa* extract

Category	Parameter	Direction of change	Dose(s) affected	Significance level
Body/Organ weight	Body weight	↓	250 mg/kg	*P*<0.05
	Pancreas wt	↓ vs control	250 mg/kg	*P*<0.01
Liver function	ALP	↓	25 mg/kg	*P*<0.05
	ALT	↓	25 & 250 mg/kg	*P*<0.05
	AST	↑	25 & 125 mg/kg	*P*<0.05–0.01
Renal/metabolic	Urea	↑	250 mg/kg	*P*<0.05
	Glucose	↑	125 & 250 mg/kg	*P*<0.01
	LDH	↑	all doses	*P*<0.01
	CK	↓	125 & 250 mg/kg	*P*<0.01
Lipids	TAG	↓	250 mg/kg	*P*<0.01
	LDL	↓	250 mg/kg	*P*<0.05
Haematology	PCV	↑	250 mg/kg	*P*<0.05
	Hb	↑	125 mg/kg	*P*<0.05
	PLT	↑	125 mg/kg	*P*<0.01
	MCHC	↓	all doses	*P*<0.05–0.01
	WBC	↓	125 & 250 mg/kg	*P*<0.05–0.01
	Lymphocytes	↓	125 & 250 mg/kg	*P*<0.01
	Neutrophils	↑	all doses	*P*<0.05–0.01
	Monocytes	↑	all doses	*P*<0.01

## Conclusion

The results of this study demonstrated the relative safety of the crude ethanol extract of *C. sativa* after consumption, with an oral LD_50_ of >5000 mg/kg body weight (GHS-Category 5, LD_50_ cut-off at 5000 mg/kg body weight) and a NOAEL dose of ≤300 mg/kg body weight. These results suggest that our indigenous variety of *C. sativa* may be considered safe for oral intake, despite the behavioral changes and significant weight loss observed in the animals. 

## Data Availability

The datasets generated and/or analysed during the current study are available from the corresponding author on reasonable request.

## Declaration

We have not used any AI tools or technologies to prepare this manuscript.

## Limitations of the Study

A notable limitation of this study was the poor solubility of the crude extract in common aqueous and organic solvents. This creates difficulties in maintaining consistent concentrations for in vitro and potential *in vivo* assays. As a result, cold-pressed coconut oil was used as the vehicle for reconstituting the crude extract.

## Availability of Data and Materials

Data will be made available upon reasonable request.

## Future Work

This research did not examine the toxicological effects of long-term use. However, it is crucial to investigate the toxic effects over extended periods (>90 days), compare toxicity profiles across different routes of exposure, and assess the impact of pharmacogenomics on toxicity, as both route and genetics are known to influence the toxicity of any substance.
