# Supplementary material for: Toxicological evaluation and preliminary phytochemical characterisation of a Nigerian Cannabis sativa chemovar
Source: Iran J Basic Med Sci. 2025;28(12):1736–42. doi: 10.22038/ijbms.2025.85494.18494 (PMC12829708; doi:10.22038/ijbms.2025.85494.18494)
Supplement: Supplementary file 1 — Supplementary Table 1. [file ijbms-28-12-1736-s001.pdf]

Supplementary Table 1. Phytoconstituents in crude *C. sativa* extract using GC-MS

Peak#	R. Time	Area	Area%	Height	Height%	A/H	Name
1	12.773	4404155	0.36	2198673	0.72	2.00	Caryophyllene
2	13.140	2956724	0.24	1224163	0.40	2.42	Humulene
3	4.432	11457532	0.95	5629124	1.85	2.04	Caryophyllene oxide
4	4.682	3613109	0.30	2087402	0.69	1.73	12-Oxabicyclo[9.1.0]dodeca-3,7-diene, 1,5,5,8-tetramethyl-, [1R-(1R*,3E
5	4.946	5543054	0.46	1476036	0.49	3.76	Tetracyclo[6.3.2.0(2,5).0(1,8)]tridecan-9-ol, 4,4-dimethyl-
6	5.129	6301076	0.52	1789832	0.59	3.52	1H-Cycloprop[e]azulen-4-ol, decahydro-1,1,4,7-tetramethyl-, [1aR-(1a.al
7	5.264	4261006	0.35	1602945	0.53	2.66	Androstan-17-one, 3-ethyl-3-hydroxy-, (5.alpha.)-
8	17.464	13217817	1.09	5235886	1.72	2.52	Hexadecanoic acid, methyl ester
9	17.879	12238442	1.01	4143543	1.36	2.95	n-Hexadecanoic acid
10	19.106	10268613	0.85	5021454	1.65	2.04	11,14-Octadecadienoic acid, methyl ester
11	19.159	16442903	1.36	6036649	1.99	2.72	9,12,15-Octadecatrienoic acid, methyl ester, (Z, Z, Z)-
12	19.233	3275047	0.27	1408219	0.46	2.33	15-Octadecenoic acid, methyl ester
13	19.375	14254194	1.18	7180053	2.36	1.99	Phytol
14	19.525	4159771	0.34	1894368	0.62	2.20	9,12-Octadecadienoic acid (Z,Z)-
15	19.582	9246968	0.77	3411072	1.12	2.71	cis,cis,cis-7,10,13-Hexadecatrienal
16	20.166	7817567	0.65	2149097	0.71	3.64	1,3-Benzenediol, 2-(3,7-dimethyl-2,6-octadecyl)-5-pentyl-$$Cannabigerol
17	20.961	7722913	0.64	3738191	1.23	2.07	1H-4-Oxabenzo(f)cyclobut(cd)inden-8-ol,1a-.alpha.,2,3,3a,8b-.alpha.,8c-$$ Cannabicyclol
18	21.202	38479211	3.18	15325562	5.05	2.51	delta.9-Tetrahydrocannabivarin
19	21.359	11455515	0.95	1542281	0.51	7.43	1H-4-Oxabenzo(f)cyclobut(cd)inden-8-ol, 1a-.alpha.,2,3,3a,8b-.alpha.,8c-$$ Cannabicyclol
20	21.708	13263853	1.10	5008820	1.65	2.65	Cannabichromene
21	21.789	23812151	1.97	7680010	2.53	3.10	6H-Dibenzo[b,d]pyran-1-ol, 6,6,9-trimethyl-3-propyl-$$ Cannabivarin
22	22.125	47344011	3.92	21776544	7.17	2.17	Cannabidiol
23	22.169	57690815	4.77	21186642	6.98	2.72	Cannabichromene
24	22.250	11229340	0.93	4175427	1.37	2.69	Terephthalic acid, dodecyl 2-methoxyethyl ester
25	22.329	23920807	1.98	7797090	2.57	3.07	2(1H)-Phenanthrenone, 3,4,4a,9,10,10a-hexahydro-7-hydroxy-1,1,4a-trim

26	22.403	20825094	1.72	5944133	1.96	3.50	Cannabicoumaronone
27	22.564	17542978	1.45	5684719	1.87	3.09	DELTA.8-Tetrahydrocannabinol
28	23.063	432339324	35.78	56311779	18.54	7.68	DELTA.9-Tetrahydrocannabinol (Dronabinol)
29	23.198	67714484	5.60	9513195	3.13	7.12	3-Methoxyestra-1,3,5(10),8,14-pentaen-17-one (+,-)-
30	23.450	117218949	9.70	17204923	5.66	6.81	1,3-Benzenediol, 2-(3,7-dimethyl-2,6-octadecyl)-5-pentyl-$$ Cannabigerol
31	23.572	68964305	5.71	34325033	11.30	2.01	Cannabinol
32	23.780	49260631	4.08	7538567	2.48	6.53	3,5-Diisopropylphenyl trifluoro methanesulfonate
33	24.230	3491423	0.29	1600718	0.53	2.18	Silane, dimethyloctyloxynonyloxy-
34	24.458	9120147	0.75	3234117	1.06	2.82	Acetyl-dihydronatalensine
35	24.574	35015787	2.90	13700782	4.51	2.56	Cannabinol
36	24.908	3119266	0.26	1032969	0.34	3.02	6H-Dibenzo[b,d]pyran-1,8-diol, 6a,7,8,9,10,10a-hexahydro-6,6-dimethyl
37	25.005	3293853	0.27	1235160	0.41	2.67	3-Heptafluorobutyryl-(-)-camphor
38	25.240	2431262	0.20	954200	0.31	2.55	Methoxy-THC
39	25.557	13618091	1.13	4713260	1.55	2.89	2-Isopropyl-5-methyl phenyl 2,2,3,3,4,4,4-heptafluorobutanoate
